# Case report: Clinical characteristics of anthrax meningoencephalitis: two cases diagnosed using metagenomic next-generation sequencing and literature review

**DOI:** 10.3389/fmed.2025.1539314

**Published:** 2025-02-12

**Authors:** Minzhe Hu, Xiaodong Qiao, Jingliang Zhang, Danqing Qin, Shougang Guo, Weili Zhao, Chunjuan Wang

**Affiliations:** ^1^Department of Neurology, Shandong First Medical University, Jinan, Shandong, China; ^2^Department of Neurology, Affiliated Hospital of Chifeng University, Chifeng, Inner Mongolia Autonomous Region, China; ^3^Department of Neurology, Shandong Provincial Hospital Affiliated to Shandong First Medical University, Jinan, Shandong, China

**Keywords:** anthrax, meningoencephalitis, metagenomic next-generation sequencing, *Bacillus anthracis*, mNGS

## Abstract

**Purpose:**

To explore the clinical features, diagnosis, treatment, and prognosis of anthrax meningoencephalitis.

**Methods:**

The clinical data of two cases of anthrax meningoencephalitis were summarized and the relevant literature was reviewed.

**Results:**

Both patients, who were farmers, had cutaneous lesions prior to the onset of meningoencephalitis. The clinical manifestations included fever (2/2), headache (2/2), stupor (2/2), meningeal signs (2/2), and lymph node enlargement (2/2). The CSF analysis showed erythrocytes, increased neutrophils, low glucose levels and high protein levels. CSF cytology revealed rod-shaped bacilli. Metagenomic next-generation sequencing of the CSF from both patients detected *Bacillus anthracis*. Additionally, cultures confirmed the presence of endogenous spores of macrobacteria. Brain imaging revealed subarachnoid hemorrhages and minimal cerebral edema. Despite aggressive antibiotic treatment, both patients died. Fifty-seven articles of the past 70 years were reviewed. There were 59 patients of anthrax meningoencephalitis in total, including 46 patients died. Stupor (42/46, 91.3% vs. 3/13, 46.2%, *p* = 0.001), agitation (15/46, 32.6% vs. 0/13, 0.0%, *p* = 0.043) and intracranial hemorrhage (37/46, 80.4% vs. 4/13, 30.8%, *p* = 0.002) were more common in the deceased group. Two types of bactericidal drugs or intrathecal injection drugs presented more often in the surviving group (10/13, 76.9% vs. 13/46, 28.3%, *p* = 0.001), whereas penicillin monotherapy presented more often in the deceased group (23/46, 50.0% vs. 2/13, 15.4%, *p* = 0.026).

**Conclusion:**

Anthrax meningoencephalitis typically presents as a rapidly progressive bacterial meningoencephalitis. The occurrence of stupor, agitation and intracranial hemorrhage is possibly correlated with poor outcome. Two types of bactericidal drugs or intrathecal injection drugs are associated with better prognosis. Metagenomic next-generation sequencing can quickly and accurately detect *B. anthracis* in CSF.

## 1 Introduction

Anthrax is a severe infectious disease caused by gram-positive, rod-shaped bacteria known as *Bacillus anthracis* (1). In 1850, Rayer discovered *B. anthracis* in the blood of infected sheep, marking the first identification of a pathogenic microorganism in human history. Over the past century, the incidence of human anthrax has declined (2). However, in recent years, there has been an increase in incidence and a wider geographical distribution of human anthrax (3). In China, human anthrax cases are predominantly reported in the western and northeastern regions, with 82% of patients coming from the provinces and autonomous regions of Sichuan, Xinjiang, Gansu, Qinghai, Guizhou, and Inner Mongolia. The incidence of the disease is higher during the summer months, while cases are relatively infrequent in the autumn and winter (4). Anthrax presents four clinical forms in humans: cutaneous, gastrointestinal, injection and inhalation (5, 6), with the majority of cases (>95%) being cutaneous (7). These lesions primarily occur on exposed areas of the body such as the hands, arms, face, and neck (8, 9).

Meningoencephalitis occurs in fewer than 5% of all anthrax patients (10), and can be a complication of all anthrax types, resulting from haematogenous or lymphatic dissemination from any primary infection across the blood brain barrier. Occasionally it may also manifest as a primary infection (11). Anthrax meningoencephalitis is a medical emergency with a high mortality rate, exceeding 95% (12). Its symptoms commonly include fever, headache, nausea, vomiting and altered mental status (13). Due to its rarity and lack of clinical specificity, many clinicians are unable to promptly diagnose anthrax meningoencephalitis based on the characteristics alone. Metagenomic next-generation sequencing (mNGS) offers a comprehensive analysis of microbial and host genetic material in clinical samples, enabling the detection of both culturable and non-culturable pathogens in the host (14, 15). mNGS serves as a valuable tool for the early and accurate identification of pathogens (1, 16). In this report, we present two cases of anthrax meningoencephalitis that were diagnosed using mNGS.

## 2 Methods and materials

### 2.1 Clinical data

#### 2.1.1 Case 1

A 49-year-old male presented with symptoms of high fever, headache and agitation lasting for 4 days, followed by a half-day period of unconsciousness. The patient, a native of Dezhou, Shandong, was engaged in the trade of wool and hides. The patient had suffered a finger injury on his left hand without proper disinfection 12 days before admission. Subsequently, he was engaged in fishing activities with his bare hands. Six days after the injury, his finger exhibited signs of discoloration, swelling, pain and warmth.

**Figure 1:**
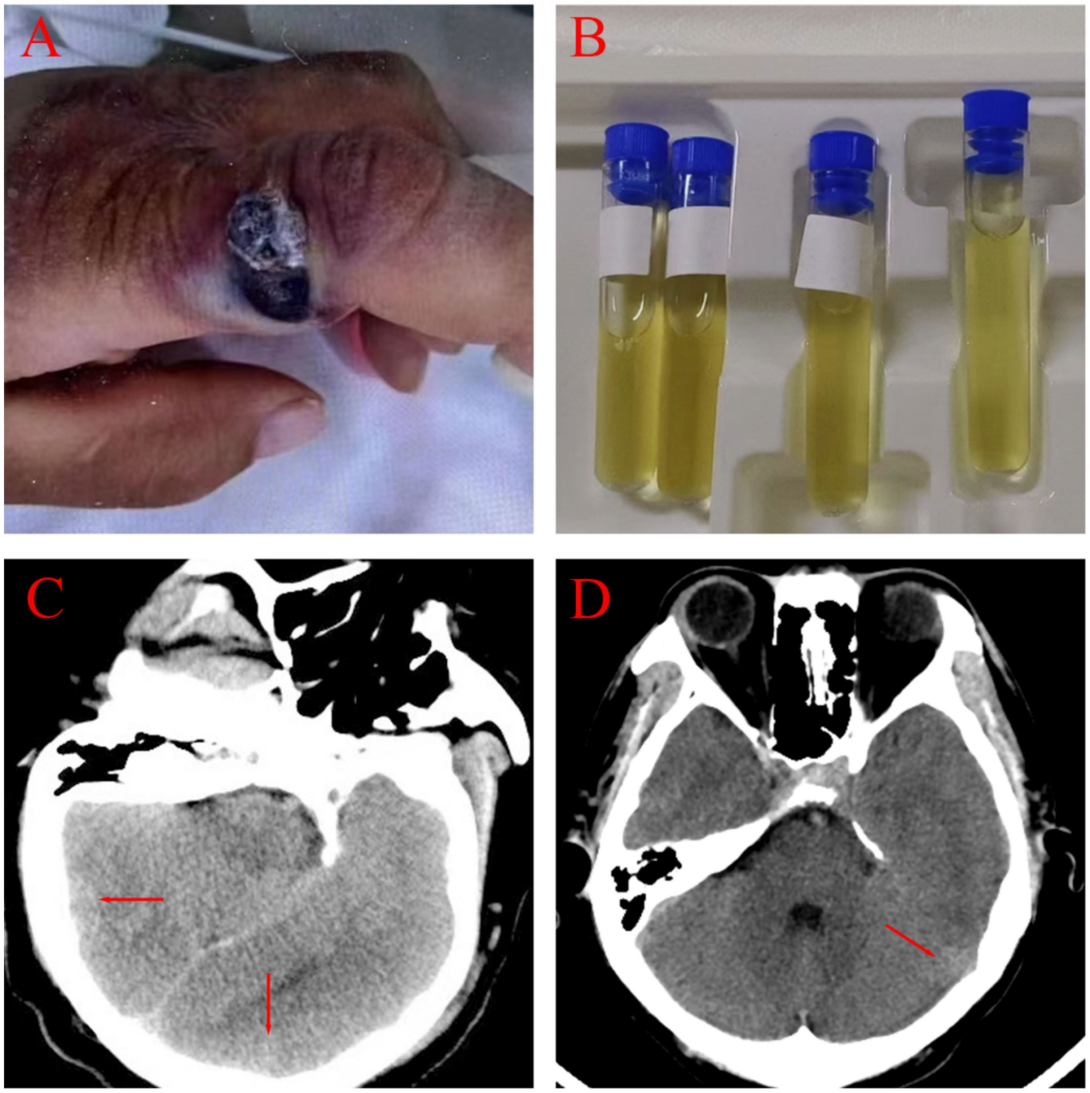
Clinical and imaging manifestations of two patients. **(A)** A round, red nodule with a central necrotic crust, raised edges, and surrounding edema was present on the index finger (Patient 1). **(B)** Visualized CSF. **(C,D)** Cranial CT findings for Patient 1 and Patient 2 showed subarachnoid hemorrhages and minimal cerebral edema.

**Figure 2:**
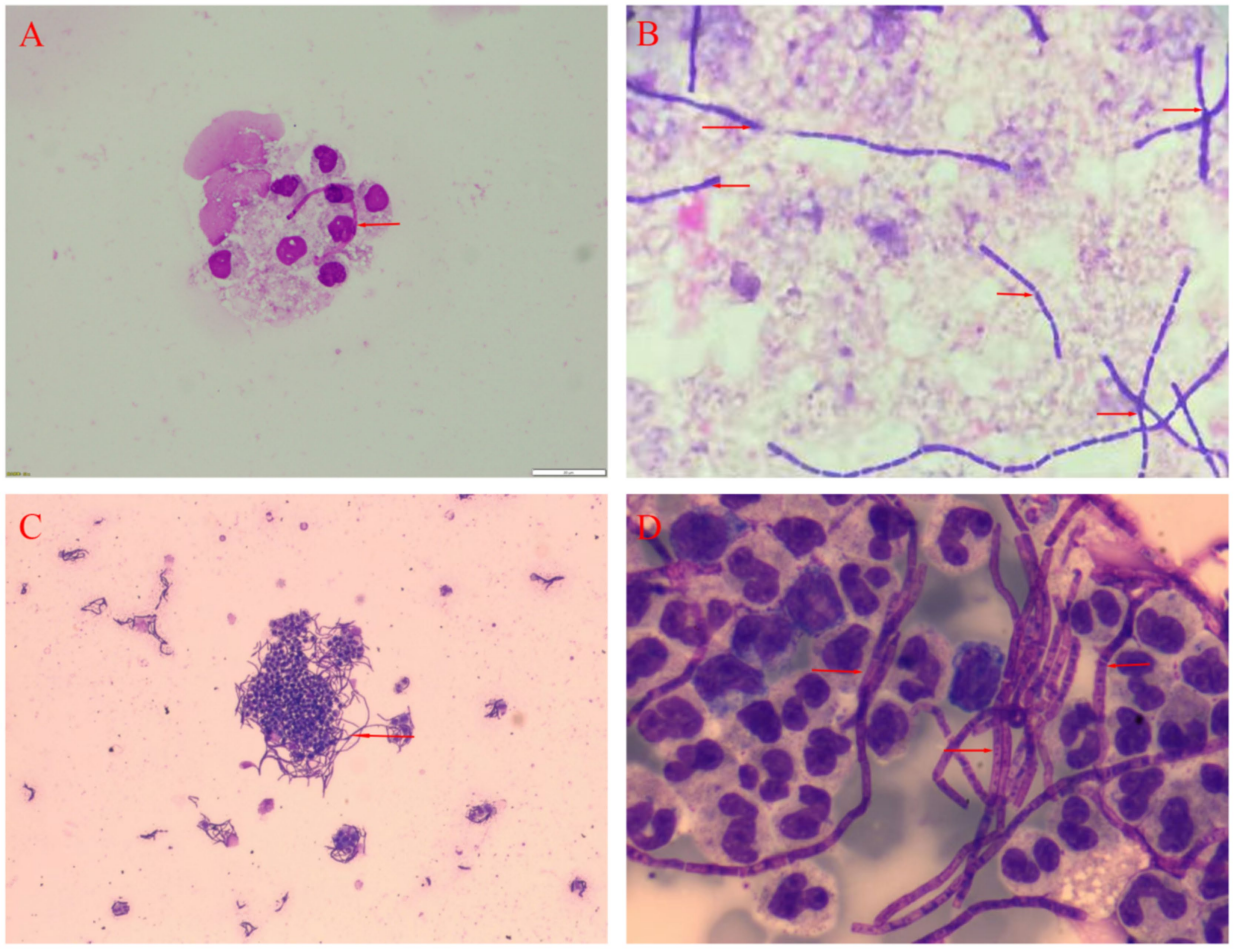
Analysis of the CSF of two patients. **(A)** Gram staining for Patient 1 *400. **(B)** Bacterial culture for Patient 1 *400. **(C)** Microscopic examination of bacterial smears for Patient 2*100. **(D)** May-Grunwald-Giemsa staining for Patient 2 *400.

On admission, the patient was febrile with a temperature of 39.8°Cand in a mild coma (Glasgow Coma Scale score of 4). A physical examination revealed a purple, swollen finger on the left hand. The surface of the finger had ulcerated, with the presence of black eschar (Figure 1A). Additionally, enlarged and tender lymph nodes were palpable in the right axilla. Neurologic examinations showed significant neck stiffness and positive Kernig’s sign bilaterally. Baseline blood counts indicated a total white blood cell (WBC) count of 16.65 × 10^9/L, with neutrophils comprising 87% of the count. Additionally, the patient exhibited elevated levels of C-reactive protein at 37.48 mg/L, procalcitonin at 0.4 ng/mL and interleukin-6 at 76.14 pg./mL. The cerebrospinal fluid (CSF) samples appeared yellow and turbid (Figure 1B), with a lumbar puncture pressure exceeding 330 mmH2O. It contained white cell count of 670 × 10^6/L, predominantly polymorphonuclear leukocytes (90%), and erythrocytes (1,000 × 10^6/L). Biochemical analysis of the CSF sample showed high protein concentration (12.38 g/L), low glucose (2.08 g/L, serum glucose 7.85 mmol/L), and a strongly positive Pan test. Capsular antigen and India ink tests were negative. Gram staining of the CSF revealed gram-positive bacilli arranged in long, bamboo-like chains with blunt, rounded, thick, and short free ends (Figure 2A). The mNGS test, completed in just 10 h, identified *B. anthracis*, accounting for 23.16% of nucleotide sequence coverage. A spread plate inoculation of the CSF was performed onto Nutrient Agar. After 24 h of incubation at 37°C in a standard incubator, the CSF culture exhibited abundant growth of endospore-forming bacilli (Figure 2B). However, blood samples for mNGS were negative. The culture of blood, wound secretion and alveolar lavage fluid all yielded negative results. The brain CT scan showed subarachnoid hemorrhages and minimal cerebral edema, with no signs of herniation (Figure 1C).

Despite receiving intravenous treatment with ceftriaxone (2 g/d) and vancomycin (1 g/12 h) during his hospitalization, the patient unfortunately died of infectious shock just four days after admission. A necropsy could not be conducted due to the lack of permission from the patient’s family. Nine months later, all his family members remained in good health, and there were no other anthrax-related deaths reported in the village where he resided.

#### 2.1.2 Case 2

The patient was a 34-year-old male, who smoked and had no known medical conditions. He was admitted to the emergency department due to fever and headache that had persisted for 4 days, along with a paroxysmal generalized tonic–clonic seizure. The patient, a farmer residing in Chifeng, Inner Mongolia Autonomous Region, owned two cattle which he personally took care of and fed. Prior to admission, his left thumb had been squeezed by a car door, and the wound was promptly sutured. The patient had cleaned cow feces and fed cattle approximately 20 days before onset. Four days prior to admission, he developed fever and headache, which worsened despite taking oral nonsteroidal anti-inflammatory drugs. Further investigation revealed that the cattle and other animals were in good health, and no similar clinical symptoms were reported in the surrounding population.

On admission, he was febrile and in a mild coma (Glasgow Coma Scale score of 8), with a temperature of 38.5°C. Physical examination revealed a bruising and swelling on his left index finger, with a visible, self-sutured wound approximately 5 centimeter in length. The wound had a moist surface and was tender to the touch. Neurologic examinations showed neck stiffness and a positive Kernig’s sign bilaterally. Baseline blood counts showed an elevated total WBC count of 14.27 × 10^9/L with 87% neutrophils. Additionally, his C-reactive protein, procalcitonin and erythrocyte sedimentation rate levels were elevated, at 197 mg/L, 57.8 ng/mL and 25 mm per hour, respectively. CSF was yellow and turbid, with a lumbar puncture pressure of 135 mmH2O. Analysis of the CSF showed a pleocytosis of 1,152 × 10^6/L, predominantly polymorphonuclears (97%), a high protein level (4.092 g/L), a low glucose level (6.76mmo/L, serum glucose 16.07 mol/L) and an high IL-6 level (66.15 pg./mL). Capsular antigen detection, ink staining and acid-fast staining of the CSF were negative. May-Grunwald-Giemsa staining of the CSF smear revealed numerous bacilli with bamboo-like segmentation (Figures 2C,D). The mNGS test confirmed the presence of *B. anthracis*. A spread plate inoculation of the CSF was performed onto Nutrient Agar. The CSF was incubated at 37°C for 24 h in a standard incubator, resulting in the growth of a significant number of spore-forming bacilli. The cranial CT scan showed some subarachnoid hemorrhages and minimal cerebral edema, with no signs of herniation (Figure 1D).

Despite being administered piperacillin sodium tazobactam (4.5 g/8 h), the patient’s condition did not improve, and he passed away two days later. The cattle and other animals were in good health, and there were no similar clinical symptoms observed in the surrounding people. Nine months later, all members of his family remained healthy, and no other anthrax-related deaths had occurred in the village where he lived.

### 2.2 Literature search and analysis

The demographic data, clinical manifestations, imaging findings, treatments and prognosis information of two anthrax meningitis patients in this study were summarized. To identify the potential causative pathogen, we employed mNGS on the Ion Torrent platform (Thermo Fisher Scientific)^1^ using DNA extracted from the CSF of the patient. DNA was extracted using the QIAamp UCP Pathogen Minikit (Qiagen, catalog number 50214, Germany). The extracted nucleic acid was then processed for library construction using the Ion Xpress™ kit, in accordance with the manufacturer’s protocol. This process involved DNA fragmentation, end repair, adapter ligation, and PCR amplification to ensure efficient library preparation. The resulting libraries were sequenced on the Ion Proton sequencer using semiconductor sequencing technology. For bioinformatics analysis, high-quality sequencing data were obtained by removing short (<50 bp), low-quality, and human-derived sequences. The remaining sequences were aligned to comprehensive pathogen databases, including bacteria, viruses, fungi, and protozoa (NCBI).^2^ Sequence analysis revealed the presence of *B. anthracis*, with a nucleotide homology of 99.8% to the reference strain *B. anthracis* (NC_007530.2) in the NCBI database. Ethics Approval Number: SWYX: NO.2024-659. Additionally, this study conducted a comparative analysis of anthrax meningoencephalitis cases retrieved from PubMed between January 1, 1952, and August 31, 2024. The data were formatted in accordance with SPSS requirements and imported into SPSS version 27.0 for statistical analysis. Categorical data were summarized using frequency statistics, and chi-square tests were applied for hypothesis testing. Count data were expressed as frequencies (percentages), and intergroup differences were compared using chi-square tests; if the chi-square test assumptions were not met, Fisher’s exact test or continuity correction was used. All statistical analyses were performed using SPSS version 27.0, with a significance level set at ɑ = 0.05 (*p* < 0.05 indicating statistical significance).

## 3 Results

### 3.1 Clinical features of two patients with anthrax meningoencephalitis

Both patients were middle-aged males who had contact with fishes or animal feces, after sustaining hand trauma prior to the onset of their illness. Their meningoencephalitis was secondary to skin infections. The clinical manifestations included fever (2/2), headache (2/2), seizure (1/2), stupor or coma (2/2), meningeal signs (2/2), lymph node enlargement (2/2), and elevated CSF pressure (1/2). Excluding puncture wounds, the CSF analysis showed the presence of erythrocytes (1/2), increased neutrophils (2/2), high protein levels (2/2), low glucose levels (1/2). CSF cytology revealed rod-shaped or filamentous bacilli. The results revealed abundant growth of spore-forming bacilli in both cultures. Pathogen mNGS of the CSF from both patients detected only *B. anthracis*. Brain imaging revealed subarachnoid hemorrhages and minimal cerebral edema. Despite aggressive anti-infective treatment, both patients succumbed to the illness approximately one week after the onset of symptoms (Table 1).

**Table 1:** Clinical data of two patients with anthrax meningoencephalitis.

	Patient 1	Patient 2
Age (years)	45	34
Sex	Male	Male
Occupation	Farmer	Farmer
Skin infection	Orbicular eschar	Open wound
Time from skin infection to meningoencephalitis symptoms	8 days	16 days
Time of onset to death of meningitis	8 days	6 days
Blood		
WBC	16.65 × 109/L (3.5 ~ 9.5 × 109)	14.27 × 109/L (3.5 ~ 9.5 × 109)
Neutrophil ratio	0.871 (0.400 ~ 0.750)	0.874 (0.400 ~ 0.750)
IL-6	76.14 pg./mL (0 ~ 5.4)	58 pg./mL (0 ~ 7)
PCT	0.4 ng/mL (0 ~ 0.05)	57.8 ng/mL (<0.046)
CRP	37.48 mg/L (0 ~ 8)	197 mg/L (0 ~ 6)
ESR	No measured	25 mm/h (0 ~ 15)
Clinical signs and symptoms		
Fever	Yes	Yes
Headache	Yes	Yes
Disturbance of consciousness	Yes	Yes
Nuchal stiffness	NO	Yes
Kernig’s sign	Yes	Yes
	Yes	Yes
CSF		
Pressure	>330mmH2O	135mmH2O
WBC	670 × 10^6/L	1,152 × 10^6/L
Neutrophil ratio	80.3%	97%
Glucose	2.08 g/L	6.76mmo/L
Chloride	111 mmol/L	105.3 mmol/L
Protein	12.38 g/L	4.092 g/L
Cytology	Rod-shaped bacilli	Rod-shaped bacilli
Culture	Endogenous spores of G+ macrobacteria	Endogenous spores of macrobacteria
mNGS	*B. anthracis*	*B. anthracis*
Cranial CT	Subarachnoid hemorrhages and minimal cerebral edema.	Normal (2d after onset of fever and headache); subarachnoid hemorrhages and minimal cerebral edema (4d after onset of fever and headache)
Blood/Wound culture	Negative	No measured
Chest radiograph	Inflammation in both lungs	Inflammation in both lungs
Antibacterial	Ceftriaxone (2 g/d) and vancomycin (1 g/12 h)	Piperacillin sodium tazobactam (4.5 g/8 h)
Clinical outcome	Died	Died

WBC, white blood cells; IL-6, interleukin 6; PCT, procalcitonin; CRP, C-reactive protein; ESR, erythrocyte sedimentation rate.

### 3.2 Analysis of prognostic factors related to anthrax meningoencephalitis

Fifty-seven articles from the past 70 years were reviewed. After excluding those with incomplete information, 57 patients were selected. Including the two patients in the study, a total of 46 patients died and 13 patients survived (Table 2). The age range of deceased patients was from 2 to 72 years (median 46), while the age range for survivors was from 6 to 70 years (median 48). Intracranial hemorrhage presented more often in deceased group than surviving group (37/46, 80.4% vs. 4/13, 30.8%, *p* = 0.002). Stupor or coma (42/46, 91.3% vs. 6/13, 46.2%, *p* = 0.001) and agitation (15/46, 32.6% vs. 0/13, 0.0%, *p* = 0.043) presented more often in deceased group than surviving group. However, fever (13/13, 100.0% vs. 29/46, 63.0%, *p* = 0.024) and headache (12/13, 92.3% vs. 24/46, 52.2%, *p* = 0.009) presented more often in surviving group than deceased group. Two types of bactericidal drugs or intrathecal injection drugs presented more often in the surviving group (10/13, 76.9% vs. 13/46, 28.3%, *p* = 0.001), whereas penicillin monotherapy presented more often in the deceased group (23/46, 50.0% vs. 2/13, 15.4%, *p* = 0.026). Additionally, no significant difference was found in terms of sex, clinical forms, respiratory symptoms, emesis, seizures, neck stiffness, Kernig’s sign, or the addition of corticosteroids to the treatment.

**Table 2:** Comparison of clinical data between surviving and deceased patients in anthrax meningoencephalitis.

Clinical characteristics	No. of patients (percentage)			*p* value
	ALL (*n* = 59)	Deceased group (*n* = 46)	Surviving group (*n* = 13)	
Median age, range (y)	46 (2–72)	46 (2–72)	48 (6–70)	
Male	46 (78.0)	35 (76.1)	11 (84.6)	0.782
Clinical forms				
Cutaneous	31 (52.5)	25 (54.3)	6 (46.1)	0.853
Gastrointestina	6 (10.2)	4 (8.7)	2 (15.4)	
Inhalationl	15 (25.4)	12 (26.1)	3 (23.1)	
Injection	3 (5.1)	2 (4.3)	1 (7.7)	
Unknow	4 (6.8)	3 (6.5)	1 (7.7)	
Respiratory symptoms	15 (25.4)	13 (28.3)	2 (15.4)	0.561
Fever	42 (71.2)	29 (63.0)	13 (100.0)	0.024*
Headache	36 (61.0)	24 (52.2)	12 (92.3)	0.009*
Emesis	25 (42.4)	18 (39.1)	7 (53.8)	0.343
Seizures	22 (37.3)	17 (37.0)	5 (38.5)	1.000
Agitation	15 (25.4)	15 (32.6)	0 (0.0)	0.043*
Stupor or Coma	48 (81.4)	42 (91.3)	6 (46.2)	0.001*
Neck stiffness	20 (33.9)	14 (30.4)	6 (46.2)	0.468
Kernig’s sign	10 (16.9)	7 (15.2)	3 (23.1)	0.804
Intracranial hemorrhage	41 (69.5)	37 (80.4)	4 (30.8)	0.002*
Treatment				
Penicillin monotherapy	25 (42.4)	23 (50)	2 (15.4)	0.026*
Two types of bactericidal drugs or intrathecal injection drugs	23 (39.0)	13 (28.3)	10 (76.9)	0.001*
Add corticosteroids	14 (23.7)	9 (19.6)	5 (38.5)	0.296

^*^p < 0.05, two groups of data have a statistically significant difference.

## 4 Discussion

Anthrax is one of the most significant zoonotic diseases, primarily affecting herbivorous animals but occasionally transmitted to humans (17, 18). The causative agent, *B. anthracis*, infects humans through contact with infected or deceased animals, contaminated animal products, or direct exposure to environmental spores (9). Anthrax meningoencephalitis, an exceedingly rare complication, typically arises as a secondary manifestation of any form of anthrax. Histopathologically, anthrax meningoencephalitis is characterized by necrotizing vasculitis, cerebral infarction, edema, hemorrhagic meningitis, intraventricular hemorrhage, and multifocal subarachnoid or parenchymal hemorrhages (10). Clinically, it presents as acute purulent meningitis, marked by abrupt onset of fever, headache, nausea, vomiting, altered mental status, focal or generalized seizures, focal neurological deficits, cranial nerve involvement, and significant meningeal irritation (13). CSF findings often reveal hemorrhagic characteristics, occasionally xanthochromic, with elevated pressure, pleocytosis, decreased glucose levels, and elevated protein concentrations (19). In our study, two patients developed rapidly progressing meningoencephalitis after hand trauma. Patient 1 sustained a hand injury while fishing. However, there is currently no evidence to indicate that fish can act as a source of anthrax infection. It is plausible that the wound might have provided a pathway for anthrax infection, probably handling wool or hides contaminated with *B. anthracis* spores, since the patient was employed in the trade of these products. Patient 1 presented with typical skin lesions of anthrax characterized by round, red nodules with central necrotic eschars, raised edges, and surrounding edema. Patient 2 did not show the characteristic eschar typically associated with cutaneous anthrax, and presumably he got infected while handling contaminated feed. This discrepancy could be attributed to early antibiotic intervention, a phenomenon also documented in similar cases from India (20).

The diagnosis of anthrax relies on a comprehensive assessment of clinical manifestations, exposure history, and microbiological confirmation of the causative agent. The gold standard for diagnosing anthrax meningoencephalitis is the isolation of *B. anthracis* from CSF (10) and the detection of its specific nucleic acids by Polymerase Chain Reaction (21). In both of our cases, CSF analysis revealed neutrophilic pleocytosis, elevated protein concentrations, and reduced glucose levels. CSF cytology disclosed rod-shaped or filamentous bacilli. However, initial species-level identification could not be definitively established. Routine culture is often negative due to the early administration of broad-spectrum or prophylactic antimicrobial agents (15). PCR typically requires specific primers and templates, which limits its use to the detection of known pathogens. Currently, traditional etiological detection methods, such as culture, immunological assays, and PCR, are limited by poor timeliness, narrow pathogen coverage, and suboptimal positive detection rates (22, 23).

In contrast, mNGS does not depend on predefined primers, enabling it to identify nearly all pathogens directly from clinical samples (1, 24–31). Moreover, mNGS is capable of detecting pathogens even in samples with minimal DNA content (14). Crucially, mNGS detects pathogens through DNA, allowing successful identification regardless of whether the microorganisms in the sample are viable or non-viable (4, 15). Moreover, mNGS boasts the capability to differentiate between infectious and non-infectious etiologies, distinguish bacterial from viral infections, and gauge infection severity by capturing infection markers via probe hybridization (32). This is especially advantageous in diagnosing rare, novel, or unknown pathogens, as well as complex infectious diseases with atypical presentations. However, the application of mNGS technology in medicine also presents certain limitations, such as complex procedures, high costs, and the absence of standardization in the detection process (33). In our two reported cases, mNGS analysis of CSF successfully identified *B. anthracis* within a mere 10 h, which significantly expedited the diagnostic process. Both patients were ultimately diagnosed with anthrax meningoencephalitis in accordance with the criteria established by the Centers for Disease Control and Prevention (34, 35).

If anthrax meningoencephalitis is suspected, empiric treatment should involve the use of three or more antimicrobial agents effective against *B. anthracis*. These should include at least one bactericidal agent, such as a fluoroquinolone or *β*-lactam, and at least one protein synthesis inhibitor, all with good CNS penetration. A combination of penicillin G or a fluoroquinolone with rifampicin is currently regarded as the first-line treatment in such cases due to its potent efficacy against *B. anthracis* and its rapid penetration into the cerebrospinal fluid (17). Treatment for anthrax meningoencephalitis should continue for a minimum of two weeks or until the patient is clinically stable. If the diagnosis cannot be ruled out, at least three weeks of combination therapy is recommended (17, 36).

Anthrax meningoencephalitis is an extremely rare condition and often fatal clinical course. Approximately 75% of patients succumb within 24 h, and overall mortality rates can reach up to 96% (37). Hemorrhagic CSF, observed in approximately two-thirds of cases, is strongly associated with poor prognosis (19). A review of reported cases of anthrax meningitis over the past 70 years has revealed that patients with anthrax meningoencephalitis who present with stupor or coma, agitation and intracranial hemorrhage have a higher mortality rate. The prognosis is more favorable when two types of bactericidal drugs or intrathecal injection drugs are used. Unfortunately, both of our patients experienced coma and hemorrhage, ultimately resulting in death.

We successfully diagnosed, for the first time, two cases of anthrax meningoencephalitis using mNGS. Prompt diagnosis and aggressive antibiotic treatment are expected to improve outcomes of anthrax meningoencephalitis.

## Data Availability

The original contributions presented in the study are included in the article/supplementary material, further inquiries can be directed to the corresponding authors.

## Footnotes

^1^ https://www.thermofisher.com

^2^ http://ftp.ncbi.nlm.nih.gov/genomes
